# The Effects of Household Air Pollution (HAP) on Lung Function in Children: A Systematic Review

**DOI:** 10.3390/ijerph182211973

**Published:** 2021-11-15

**Authors:** Sathya Swarup Aithal, Shireen Gill, Imran Satia, Sudhir Kumar Tyagi, Charlotte E. Bolton, Om P. Kurmi

**Affiliations:** 1 The Global Health Office, McMaster University, Hamilton, ON L8S 4K1, Canada; aithalss@mcmaster.ca (S.S.A.); gills14@mcmaster.ca (S.G.); 2 Department of Medicine, Division of Respirology, McMaster University, Hamilton, ON L8S 4K1, Canada; satiai@mcmaster.ca; 3 Firestone Institute for Respiratory Health, St. Joseph’s Healthcare, McMaster University, Hamilton, ON L8N 4A6, Canada; 4 Department of Energy Science and Engineering, Indian Institute of Technology, New Delhi 110016, India; sudhirtyagi@yahoo.com; 5 NIHR Nottingham BRC Respiratory Theme, School of Medicine, University of Nottingham, Nottingham NG7 2UH, UK; charlotte.bolton@nottingham.ac.uk; 6 Faculty Centre for Intelligent Healthcare, Coventry University, Coventry CV1 5FB, UK; 7 Nexus Institute of Research and Innovation, Lalitpur 44700, Nepal

**Keywords:** household air pollution, lung function, solid fuels, indoor pollution

## Abstract

The World Health Organization (WHO) estimates that around 3 billion people today are exposed to smoke from the household combustion of solid fuels. While the household use of solid fuels has decreased over the last few decades, it remains a leading modifiable risk factor for the global burden of disease. This systematic review analyzed the impact of Household Air Pollution (HAP) on lung function in children (under 18 years of age), as this is the time period of accelerated growth rate until full skeletal maturity. Data from 11 published studies demonstrated that exposure to smoke from solid fuel was associated with a lower growth rate of several lung function indices (FVC, FEV_1_, FEF_25–75_) in children. However, there was no observed association between HAP and the FEV_1_/FVC ratio over time. Although the evidence suggests an inverse association between high exposure to HAP and lung function indices, there is a lack of longitudinal data describing this association. Therefore, precaution is needed to reduce the smoke exposure from solid fuel burning.

## 1. Introduction

The World Health Organization (WHO) estimates that around 3 billion people or about 40% of the world’s population are exposed to smoke from the burning of solid fuel (coal, wood, animal dung, or crop waste) burning for cooking or heating purposes [1]. The combustion of these solid fuels is inefficient and produces high airborne pollutants, including soot particles that can penetrate the lungs. [2] The incomplete combustion or inefficient combustion of these fuels emit smoke containing high levels of pollutants such as carbon monoxide, oxides of nitrogen, and sulphur, which are detrimental to human health. This has been linked to impaired lung function and respiratory morbidities such as asthma and lower respiratory tract infections [2,3]. Despite substantial reduction in the use of solid fuels globally, exposure to Household Air Pollution (HAP) from using these fuels for cooking remains a leading modifiable risk factor for global disease burden [4]. Among environmental risk factors, the contribution of HAP to disease burden is second only to ambient particulate matter pollution. In 2019, 91.5 million global disability-adjusted life years (DALYs) were attributable to HAP, a decline of more than 50% from 1990; however, the absolute number exposed to HAP has remained the same over the last four decades [5]. In total, 2.31 million global deaths were attributable to HAP and accounted for 4% of all deaths in 2019. The HAP-attributable burden remains the highest in sub-Saharan Africa and South Asia, with 3770.3 and 2068.0 age-standardized DALYs per 100,000 population, respectively [4,5]. Additionally, the poorest countries from low-income and middle-income countries (LMICs) are associated with the highest prevalence of HAP related complications.

People, particularly women and children in LMICs, spend a considerable amount of their time indoors, with poor ventilation systems making them more susceptible to HAP. HAP accounts for two million yearly deaths from Acute Respiratory Infections (ARI) in children [1]. Children may be especially vulnerable to indoor pollutants because of their immature immune systems and at a time period of rapid growth and development. Infants and children also inhale a larger dose of air per unit of body mass at a given activity level than adults in the same environment, hence, inhaling disproportionately high concentrations of air pollutants [2].

Timely and accurate information is urgently needed to facilitate the development of effective global health strategies to prevent further damage to the lung from HAP. There have been a limited number of studies investigating the relationship between exposure to HAP and lung function impairment in children. Our primary aim was to systematically summarize, synthesize, and analyze the extent of HAP-related lung impairment in a pediatric population from peer-reviewed publications.

## 2. Materials and Methods

This systematic review was conducted in accordance with the Preferred Reporting Items for Systematic Reviews and Meta-Analyses (PRISMA) guidelines. An a priori protocol was published in PROSPERO, ID: CRD42021236671 [6].

The following databases were systematically searched from 1980 to 21 February 2021, to identify studies: Ovid EMBASE, MEDLINE, the Global Health, Web of Science, and Scopus. Variations of the terms “air pollution”, “lung function”, and “children” were used with the “AND” Boolean operator. A complete list of keywords and search strategies is attached as Table S1. Following the predetermined inclusion criteria, titles/abstracts and full texts of retrieved articles were independently screened by two reviewers (S.A. and S.G.). Any arising conflicts were resolved by a third reviewer (O.K.). The bibliographic reference lists from studies selected for inclusion were manually checked for potential inclusion by a reviewer (S.A).

Original articles written in English were included if they involved human participants less than or equal to 18 years of age, recorded exposure to HAP, and measured lung function, with a comparator group of exposure. Participants exposed to HAP from occupational exposures were ineligible for inclusion. Studies investigating sources of air pollution that were exclusively outdoor/ambient, non-fuel combustion, tobacco smoke exposure, allergens, or those that did not distinguish between outdoor and household air pollution were excluded.

Randomized controlled trials, cohort studies, case-control studies, cross-over studies, and cross-sectional studies were included in this review. Grey literature and case reports/series were excluded from this review. Additionally, conference abstracts, posters and studies with irretrievable full texts were excluded. Lastly, studies that lacked a comparator group were not considered for inclusion.

Two reviewers (S.A. and S.G.) independently extracted data from each included study. Data collected included study design, country, participant characteristics, fuel exposure type and comparator, outcomes, ascertainment of outcomes, and data required for risk of bias assessment. HAP was defined as indoor air pollution from domestic solid-fuel combustion for cooking and/or heating (wood, charcoal, kerosene, animal dung, crop residues, pellets, coke, and coal). Pulmonary function, measured by volumes or flow rates during spirometry, was recorded in those exposed to HAP. Outcomes included metrics for pulmonary function, which were Forced Vital Capacity (FVC), Forced Expiratory Volume in 1 s (FEV_1_)**,** the ratio of FVC and FEV_1_ (FVC/FEV_1_), Forced mid-expiratory flow (FEF_25–75_), and Peak Expiratory Flow Rate (PEFR). The comparator group was defined as children exposed to relatively cleaner household fuels (such as Liquified Petroleum Gas (LPG), natural gas, or electricity) or a non-exposed comparator group.

The Risk of Bias (ROB) Assessment was conducted using the Newcastle Ottawa scale (NOS) by two independent investigators (S.A) and (S.G). Cross-sectional studies’ ROB was assessed using a modified Newcastle-Ottawa scale. The ‘star system’ used in the NOS judged a study based on three broad perspectives: the selection of the groups of study (maximum of 4 stars); the ascertainment of the exposure or outcome of a study in case-control or cohort studies, respectively (maximum of 3 stars), and the comparability between the groups of study (maximum of 2 stars). A study could therefore have a maximum of 9 stars. Conflicts were resolved by a third reviewer (O.K). The ROB assessment for individual studies can be found in Tables S2 and S3 for cohort and cross-sectional studies, respectively.

We did not conduct a meta-analysis due to high heterogeneity in study design and between the subjects in the included studies in age, geographic location, race, and factors such as the outcome assessment. Instead, the effect estimates were summarized by grouping according to lung function outcome. The range, distribution, and direction of HAP on each specified measure of lung function, FVC, /FEV_1_, FEV_1_/FVC, FEF_25–75_, and PEF was organized. The metrics used for synthesis and interpretation included measures of effect such as percent predicted values, mean values, and mean differences.

## 3. Results

### 3.1. Study Characteristics

**Figure 1:**
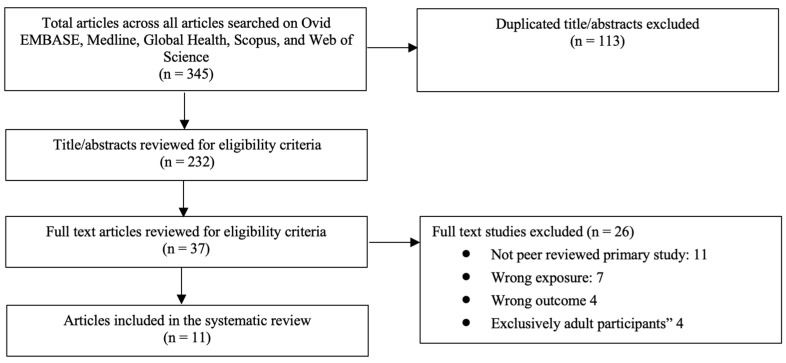
PRISMA flow diagram.

The initial search yielded 345 results. After removing duplicates, 232 titles and abstracts were screened, following which a further 195 articles were excluded as they did not meet the eligibility criteria. Of the remaining 37 articles, which moved on to the full-text screening stage, 11 were included in the systematic review and used for the data extraction and analysis. The PRISMA flow diagram in Figure 1 below illustrates the search results. In total, three cohort studies and eight cross-sectional studies were included. The studies included were published between the years 1990 and 2016. Three studies were conducted in low-income countries, six were conducted in upper-middle-income countries, and one was conducted in a high-income country. Specifically, the included studies took place in Brazil [7], China [8], Ecuador [9], Guatemala [10], Turkey [11], Honduras [12], India [13], Jordan [14], Malaysia [15], Nigeria [16], and Poland [17]. All included studies had a score of ≥6 and were considered high quality based on the Newcastle-Ottawa Scale. A summary of the included studies can be found in Table S4.

### 3.2. Population Characteristics

In total, data were collected from eleven studies consisting of a total of 10,590 participants. The ages of participants ranged from 5 to 17 years. Of the studies that provided the sex distribution of participants, the percentage of males ranged from 45.1% to 57.9%. The exposures included the use of fuels for household heating and/or cooking such as coal [8,17], wood [14,15], biomass [7,9,10,12,13,16,18], and natural gas or electricity [9,13].

### 3.3. Study Outcomes

All eleven of the included studies provided spirometry measurements of lung function. FEV_1_, FVC, FEV_1_/FVC, FEF_25–75_, and PEF were the frequently reported lung function outcomes.

#### 3.3.1. FEV_1_

The use of coal was associated with a 16.5 mL/year (33% of average annual growth rate of FEV_1_, *p* < 0.001) lower annual growth of FEV_1_ when compared to those with no use of coal in a prospective cohort study of 3273 children aged 6–13 years [8]. Similarly, a prospective cohort study of 506 Guatemalan children aged 5–8, exposed to biomass combustion reported a decrease of 44 mL/year (*p* = 0.07) in annual FEV_1_ growth among those with a chimney-stove (named *Plancha*) intervention at 18 months, compared to those with the chimney-stove installation at birth [10]. The effects of postnatal exposure to HAP on preadolescent lung function were also examined by a retrospective cohort study from Poland that found that increased exposure to indoor pollution in the winter during the first six months of life was inversely related to FEV_1_ (*β* = −0.13, *p* = 0.03) [17], when measured at 9 years of age, for 1036 children. A cross-sectional study of Ecuadorian children aged 7–15 years reported that those living in homes with the exclusive use of biomass for cooking were associated with lower FEV_1_ than those using LPG exclusively. (*β* = −0.39, *p* < 0.01) [9]. Similarly, the use of biomass for cooking was associated with a lower mean FEV_1_ of 80 mL among Malaysian children aged 7–15 [15]. In India, a study reported children living in households that used biomass for cooking had a mean FEV_1_ of 0.6 L/s (*p* = 0.005) lower than those using LPG [13].

#### 3.3.2. FVC

A prospective cohort study of 3273 children aged 6–13 years from China reported that the lifetime use of coal as household heating fuel was associated with a mean decrease of 20.5 ml/year in FVC growth over four years compared with those who had no use of coal as a household heating fuel (39% of average annual growth rate of FVC, *p* < 0.001) [8]. Similarly, a prospective cohort study of 506 children from Guatemala aged 5–8 years demonstrated a large but statistically nonsignificant decrease in FVC growth of 39 mL/year (*p* = 0.16) among those with biomass combustion and chimney-stove intervention at 18 months, compared with those with stove installation at birth [10]. The effects of postnatal exposure to HAP on preadolescent lung function were also examined in a retrospective cohort study from Poland that found that increased exposure to indoor pollution in the winter during the first six months of life was inversely related to FVC (*β* = −0.15, *p* = 0.01), measured at age 9 [17]. Cross-sectional studies showed similar lower mean FVC values in children exposed to the combustion of solid fuels. A cross-sectional study of children from Ecuador aged 7–15 years reported that those living in homes with the exclusive use of biomass for cooking had lower FVC than those using LPG exclusively. (*β* = −0.41, *p* < 0.05) [9]. Similarly, the use of biomass for cooking was associated with a lower mean FVC of 90 mL among children from Malaysia aged 7–15 years [15]. In India, a study reported children living in households that used biomass for cooking had a mean FVC of 1.7 L/s (*p* = 0.002) lower than those using LPG [13]. The studies that reported percent predicted values of FVC showed decreases between 5 and 15% with exposure to biomass combustion [15,18]

#### 3.3.3. FEV_1_/FVC

A prospective cohort study of children aged 6–13 years reported no significant longitudinal changes in FEV_1_/FVC ratio were observed in association with coal use or ventilation [8]. Similarly, the other cohort and cross-sectional studies examining the effect of HAP on FEV_1_/FVC among children reported no differences between different categories of solid fuel use.

#### 3.3.4. FEF_25–75_

A prospective cohort study of 506 children from Guatemala aged 5–8 years reported a lower statistically nonsignificant FEF_25–75_ annual growth rate (−23 mL/min/year, *p* = 0.73) among chimney cookstove installation at 18 months, compared to those with installation at birth [10]. A cross-sectional study of 1905 children from Jordan aged 7–15 years reported that those with lifetime exposure to wood and kerosene stoves had a mean unadjusted FEF_25–75_ of 0.62 L/s (*p* < 0.005) lower than those without lifetime exposure to wood and kerosene stoves [14]. Similarly, a cross-sectional study of 77 children from Ecuador aged 7–15 years reported that those living in homes that the use of biomass primarily for cooking was associated with lower FEF_25–75_ when compared to LPG as well (*β* = −0.89, *p* < 0.05) [9].A large cross-sectional study of 1505 children from rural east India aged 5–10 years showed that those living in homes that used biomass cooking fuel exclusively had a lower FEF_25–75_ of 0.77 L/s (*p* = 0.012) than those using LPG [13].

## 4. Discussion

The study results demonstrate that the use of solid fuels may lower the lung volumes and flow rates in children (FVC, FEV_1_, FEF_25–75_). There was no observed association between HAP and the FEV_1_/FVC ratio over time. This would suggest that HAP mainly brought similar reductions in FEV_1_ and FVC. However, the findings are based on a few studies only and would need to be validated from large prospective cohort studies. To the best of our knowledge, this is the first systematic review to examine the association of HAP on lung function in children.

There is a need for more prospective studies to assess the long-term impacts and exposure–response nature of the risk of HAP on lung development and function. There is also a need for further research into the effects of solid fuel combustion on the lung development of children under the age of 5 years. It is known that the first two years of life are vital for the development of the lungs [19]. However, there has been very little research examining the association between solid fuel combustion and lung function at this age to the best of our knowledge. This is because it is hard to assess lung function in children under the age of 5 years, and it is due also to the unavailability of resources including the spirometers in rural areas of LMICs where HAP is predominantly used. However, oscillometry is increasingly being used in pulmonary clinical practice, as well research to assess lung function in such populations [20]. Given the higher risk faced by women and children, the impacts of HAP on sex and gender should be a consideration in future research examining this association.

There is a possibility of residual confounding factors in the association between solid fuel use and lung function. For instance, socioeconomic factors such as income, education, and nutrition could lead to changes in health status, lung growth, and the greater use of solid fuels. In studies that encompass a variety of socioeconomic backgrounds (for instance, studies including rural and urban participants), adjustment for socioeconomic status is necessary. Questionnaire responses to solid fuel use, such as the choice of fuel and frequency of use, can lead to exposure misclassification and bias towards the null. It is also probable that the parents of children with respiratory ailments could have over-reported or under-reported the duration of use of solid fuels. Basic spirometry can be measured by the investigator with relative ease and inexpensive equipment. However, interpretation of spirometry tests can be challenging, as the tests are primarily dependent on participant effort and cooperation. Therefore, prior to interpretation, spirometry results must always be assessed for validity, which may not have been conducted in every study. The context of a spirometry test is important, as these values can vary between height, weight, age, sex, or ethnic background.

The methodological quality of the studies included in the present review was relatively homogenous, as determined by the NOS tool. However, the NOS tool is designed for classic cohort and case-control studies. Properly assessing the quality of environmental cross-sectional studies remains a challenge. To avoid selection bias, two independent reviewers conducted the screening and selection processes. However, some unpublished studies and studies not in English were not included. Therefore, there is a chance of publication bias. However, the included studies were diverse in their geographic area of study.

The scope of the current review was limited to the childhood population. However, diminished lung function in childhood is a strong predictor of subsequent low lung function in adulthood [19,21,22]. This may in turn predispose the affected to develop diseases such as COPD in adulthood [23]. However, this study demonstrates the harmful effects of HAP on lung function in children and demonstrates the need for policy to enact early intervention.

## 5. Conclusions

This systematic review contributes to the evidence of the adverse impact of HAP on lung function in children. The results of this study suggest that the household use of solid fuels such as coal, wood, kerosene, and biomass may significantly lower lung function growth (FVC, FEV_1,_ and FEF_25–75_) among children.

## Data Availability

No additional data available.

## Supplementary Materials

The following are available online at https://www.mdpi.com/article/10.3390/ijerph182211973/s1, Table S1: Search Strategy, Table S2: Risk of Bias analysis using the Newcastle Ottawa Scale for cohort studies, Table S3: Risk of Bias analysis using the modified Newcastle Ottawa Scale for cross-sectional studies, Table S4: Summary of studies included.
