# Supplementary material for: The Effects of Household Air Pollution (HAP) on Lung Function in Children: A Systematic Review
Source: Int J Environ Res Public Health. 2021 Nov 15;18(22):11973. doi: 10.3390/ijerph182211973 (PMC8623006; doi:10.3390/ijerph182211973)
Supplement: Supplementary file 1 [file ijerph-18-11973-s001.zip › ijerph-1457012-supplementary.pdf]

Table S1. Search Strategy employed to search for studies examining the impact of HAP on lung function in children. Databases searched include OVID EMBASE, MEDLINE, Global Health, Scopus, AND Web of Science.

#	Searches
1	Air Pollution, Indoor/
2	((indoor or household or house hold or home or inside) adj2 air pollution).mp. [mp=title, abstract, heading word, drug trade name, original title, device manufacturer, drug manufacturer, device trade name, keyword, floating subheading word, candidate term word]
3	((indoor or household or house hold or home or inside) adj5 air pollution).mp.
4	or/1-3
5	Coal/
6	biomass/
7	biomass.mp.
8	charcoal/
9	charcoal.mp.
10	kerosene/
11	kerosene.mp.
12	plant residue/
13	(crop residue or plant residue).mp.
14	((coal or peat or wood or dung or charcoal or biomass or kerosene or crop residue) adj5 (cook* or heat* or stove* or burn* or combust* or furnace)).mp.
15	or/5-14
16	adolescent/ or exp child/ or infant/
17	(child* or infan* or adolescen* or teenage* or preadolescen* or preteen* or preschool* or youth*).mp.
18	Pediatrics/
19	p?ediatric*.mp.
20	or/16-19

31	exp spirometry/
32	exp lung function/
33	exp forced expiratory volume/
34	exp peak expiratory flow/
35	exp forced vital capacity/
36	(lung function or spirometr* or forced expiratory volume or peak expiratory flow or forced vital capacity or FEV1 or FVC or PEF or FEF).mp.
37	or/31-36
38	4 and 15 and 20 and 37

Table S2. Risk of Bias analysis using the Newcastle Ottawa Scale for cohort studies.

Source	Selection				Comparability based on design and analysis	Outcome			Total
	Representativeness of the exposed cohort	Selection of the non-exposed cohort	Ascertainment of exposure to HAP	Demonstration of absence of outcome of interest at start		Assessment of outcome	Length of follow-up	Adequacy of follow-up	
Heinzerling et al., 2016 [9]	+	+	+	-	++	+	+	+	8
Roy et al., 2012 [7]	+	+	-	-	++	+	+	+	7
Jedrychowski et al., 2005 [16]	+	+	+	+	++	+	+	+	9

Table S3. Risk of Bias analysis using the modified Newcastle Ottawa Scale for cross-sectional studies.

Source	Selection				Comparability based on design and analysis	Outcome		Total
	Representativeness of the sample	Sample size	Non-respondents	Ascertainment of exposure to HAP		Assessment of outcome	Statistical test	
Azizi & Henry, 1990 [14]	+	+	-	-	++	++	+	7
Gharaibeh, 1995 [13]	+	+	-	+	+	++	+	6
Oluwole et al., 2013 [15]	+	-	-	+	+	++	+	6
Padhi & Padhy, 2008 [12]	+	+	-	-	++	++	+	7
Rinne et al., 2006 [8]	+	+	-	-	++	++	+	7
Thacher et al., 2013 [17]	+	+	-	+	-	++	+	6
Da Silva et al., 2012 [6]	+	+	-	+	++	+	+	7
Rennert et al., 2015 [11]	+	+	-	+	++	++	+	9

Table S4. Summary of included studies.

First Author, year	Title	Journal	Region of Study	Type of Study	Age	n	Objective of Study	Selected Outcomes	Risk of Bias Assessment (NOS scale)
*Azizi & Henry, 1990 [14]	Effects of indoor air pollution on lung function of primary school children in Kuala Lumpur	Pediatric Pulmonology	Malaysia	Cross-sectional study	7-12y	1414	We examined the relationships between exposure to indoor environmental factors, namely mosquito repellents, environmental tobacco smoke, and cooking stoves, and levels of lung function in Malaysian children.	PPV, and mean values of FVC, FEV_1, FEF_{25-75} and PEFR	7
*Da Silva et al., 2012 [6]	Impaired lung function in individuals chronically exposed to biomass combustion	Environmental Research	Brazil	Cross Sectional study	9-10y	429	To evaluate the respiratory effects of biomass combustion and compare the results with those of individuals from the same community in Brazil using liquefied petroleum gas	Mean values a FEV_1/FVC and precent predicted values of FEV_1.	7
*Gharaibeh, 1995 [13]	Effects of indoor air pollution on lung function of primary school children in Jordan	Annals of Tropical Paediatrics	Jordan	Cross Sectional study	7-13y	1905	To determine the effect of environmental exposures to unvented cook stoves on respiratory function.	mean values of spirometric measures - FVC, FEV1, FEF25-75, and PEFR	6
*Heinzerling et al., 2016 [9]	Lung function in woodsmoke-exposed Guatemalan children following a chimney stove intervention	Thorax	Guatemala	Prospective cohort study	5-8y	506	To determine the effect of early childhood HAP exposure on growth of lung function with a chimney stove intervention	PPV, mean values of spirometric measures - FEV_1, FVC, FEV_1/ FVC, and FEF_{25-75} and PEF	8
*Jedrychowski et al., 2005 [16]	Effect of indoor air quality in the postnatal period on lung function in pre-adolescent children: A retrospective cohort study in Poland	Journal of the Royal Institute of Public Health	Poland	Cohort Study	9y	1036	The purpose of this study was to determine the association between level of lung function in pre-adolescence and indoor air quality in the postnatal period.	Mean values and regression coefficients of spirometric measures – FVC, and FEV_1.	
*Oluwole et al., 2013 [15]	Effect of stove intervention on household air pollution and the respiratory health of women and children in rural Nigeria	Global Journal of Health Science	Nigeria	Cross sectional study	6-17y	59	The objective of the study was to investigate the extent of household air pollution from biomass fuels and the effectiveness of stove intervention to improve indoor air quality, exposure-related health problems, and lung function	PPV, mean values of spirometric measures - FVC, FEV_1, FEF_{25-75}, and PEFR	6
*Padhi & Padhy, 2008 [12]	Domestic fuels, indoor air pollution, and children's	Annals of the New York	India	Cross sectional study	5-10y	1505	To investigate the association between household use of	mean values of PEF, FVC, FEV_1,	7

	health: The case of rural India	Academy of Sciences					biomass	FEV_1/FVC, $FEF_{25\text{-}75\%}$,	
*Rennert et al., 2015 [11]	The effects of smokeless cookstoves on peak expiratory flow rates in rural Honduras	Journal of Public Health	Honduras	Cross Sectional study	6-14y	87	To assess the effects of improved stove designs on the peak expiratory flow rates and respiratory health of community members	Mean values of spirometric measurements - PEFR	9
*Rinne et al., 2006 [8]	Relationship of pulmonary function among women and children to indoor air pollution from biomass use in rural Ecuador	Respiratory Medicine	Ecuador	Cross Sectional study	7-15y	77	To examine the impact of biomass fuel use on pulmonary function among women and children in a rural Ecuadorian community.	Mean value of spirometric measures – FVC, FEV_1, FEV_1/FVC, $FEF_{25\text{-}75}$	7
*Roy et al., 2012 [7]	Indoor air pollution and lung function growth among children in four cities in China	Indoor Air	China	Prospective cohort study	6-13y	3273	The current analyses focused on examining the relationship of children's lung function growth with household coal burning and household ventilation practices.	Mean value of spirometric measures – FVC, FEV_1, and FEV_1/FVC	7
*Thacher et al., 2013 [17]	Biomass fuel use and the risk of asthma in Nigerian children	Respiratory Medicine	Nigeria	Cross Sectional study	5-11y	299	We studied the relationship of biomass fuel use with asthma symptoms and lung function in Nigerian children.	PPV, mean values of spirometric measures – FEV_6, FEV_1, FEV_1/ FEV_6	6
